# Supplementary material for: Phenotypical Characterization of C9ALS Patients from the Emilia Romagna Registry of ALS: A Retrospective Case–Control Study
Source: Genes (Basel). 2025 Mar 4;16(3):309. doi: 10.3390/genes16030309 (PMC11942173; doi:10.3390/genes16030309)
Supplement: Supplementary file 1 [file genes-16-00309-s001.zip › genes-3477927-supplementary.pdf]

Table S1. Univariate and multivariate analysis for female and male C9ALS patients.

	Female C9ALS				Male C9ALS			
Variable	Univariate analysis		Multivariate analysis		Univariate analysis		Multivariate analysis	
	HR (95% CI)	P value	HR (95% CI)	P value	HR (95% CI)	P value	HR (95% CI)	P value
**Family history for ALS, presence**	1.46 (0.64–3.31)	0.371			0.52 (0.23–1.18)	0.117		
**Family history for other NDD, presence**	0.87 (0.35–2.13)	0.753			1.46 (0.60–3.60)	0.406		
**Psychiatric family history, presence**	0.60 (0.17–2.07)	0.416			1.48 (0.43–5.05)	0.534		
**Age at onset, years**	1.00 (0.96–1.05)	0.851			1.02 (0.98–1.06)	0.421		
**Diagnostic delay, months**	0.92 (0.86–0.99)	0.034			0.92 (0.84–1.00)	0.052		
**BMI at diagnosis, kg/m²**	1.04 (0.98–1.11)	0.176			1.02 (0.89–1.16)	0.788		
**Weight loss, % of healthy weight**	1.03 (0.98–1.08)	0.233			1.14 (1.05–1.24)	0.001	**1.16 (1.02–1.33)**	**0.024**
**Onset, bulbar**	1.01 (0.44–2.33)	0.987			0.81 (0.35–1.90)	0.634		
**ALSbi, presence**	1.68 (0.70–4.01)	0.245			4.10 (1.32–12.73)	0.015		
**ALSci, presence**	1.79 (0.73–4.36)	0.200	3.87 (1.12–13.34)	**0.032**	4.80 (1.61–14.29)	0.005		
**FVC at diagnosis, %**	0.99 (0.97–1.01)	0.356			0.98 (0.96–1.01)	0.156		
**ALSFRS-r at diagnosis, points**	0.93 (0.84–1.02)	0.128			0.98 (0.89–1.08	0.665		
**Disease progression rate at diagnosis, points/month**	1.70 (1.20–2.41)	0.003	5.06 (2.05–12–48)	**<0.001**	1.25 (0.99–1.57)	0.064		
**Depression, presence**	0.92 (0.36–2.33)	0.853			0.53 (0.15–1.84)	0.313		
**Psychosis, presence**	3.82 (1.10–13.30)	0.035	8.23 (1.12–60.75)	**0.039**				
**Chronic Obstructive Pulmonary Disease (COPD), presence**	4.38 (0.54–35.64)	0.167			3.74 (0.46–30.41)	0.218		
**Diabetes, presence**	1.08 (0.25–4.70)	0.923						
**Cardiopathies, presence**	1.14 (0.27–4.93)	0.858			1.89 (0.63–5.67)	0.258		
**Hypertension, presence**	1.53 (0.65–3.60)	0.332			1.04 (0.44–2.43)	0.937		
**Dyslipidemia, presence**	1.28 (0.47–3.47)	0.634			0.48 (0.16–1.47)	0.200		
**Autoimmune Diseases, presence**	1.65 (0.55–4.94)	0.369			0.53 (0.07–4.02)	0.541		
**Cancer history, presence**	1.17 (0.42–3.24)	0.760			3.18 (0.38–26.74)	0.287		
**Previous trauma, presence**	0.86 (0.28–2.64)	0.792			2.46 (0.91–6.69)	0.077		
**Former tobacco smoking**	1.22 (0.48–3.11)	0.681			0.90 (0.30–2.74)	0.854		
